# Diverging trends in educational inequalities in cancer mortality between men and women in the 2000s in France

**DOI:** 10.1186/1471-2458-13-823

**Published:** 2013-09-10

**Authors:** Gwenn Menvielle, Grégoire Rey, Eric Jougla, Danièle Luce

**Affiliations:** 1 Inserm U1018, Center for Epidemiology and Population Health, Occupational and social determinants of health, Bat 15/16 Hôpital Paul Brousse, 16 ave Paul Vaillant Couturier, Villejuif Cedex 94807, France; 2 University of Versailles Saint Quentin, UMRS 1018, France; 3 Inserm, CépiDc, Le Kremlin-Bicêtre, France; 4 Inserm U1085, Irset, Pointe-à-Pitre, Guadeloupe, French West Indies

**Keywords:** Cancer mortality, France, Men, Women, Education, Time trends

## Abstract

**Background:**

Socioeconomic inequalities in cancer mortality have been observed in different European countries and the US until the end of the 1990s, with changes over time in the magnitude of these inequalities and contrasted situations between countries. The aim of this study is to estimate relative and absolute educational differences in cancer mortality in France between 1999 and 2007, and to compare these inequalities with those reported during the 1990s.

**Methods:**

Data from a representative sample including 1% of the French population were analysed. Educational differences among people aged 30–74 were quantified with hazard ratios and relative indices of inequality (RII) computed using Cox regression models as well as mortality rate difference and population attributable fraction.

**Results:**

In the period 1999–2007, large relative inequalities were found among men for total cancer and smoking and/or alcohol related cancers mortality (lung, head and neck, oesophagus). Among women, educational differences were reported for total cancer, head and neck and uterus cancer mortality. No association was found between education and breast cancer mortality. Slight educational differences in colorectal cancer mortality were observed in men and women. For most frequent cancers, no change was observed in the magnitude of relative inequalities in mortality between the 1990s and the 2000s, although the RII for lung cancer increased both in men and women. Among women, a large increase in absolute inequalities in mortality was observed for all cancers combined, lung, head and neck and colorectal cancer. In contrast, among men, absolute inequalities in mortality decreased for all smoking and/or alcohol related cancers.

**Conclusion:**

Although social inequalities in cancer mortality are still high among men, an encouraging trend is observed. Among women though, the situation regarding social inequalities is less favourable, mainly due to a health improvement limited to higher educated women. These inequalities may be expected to further increase in future years.

## Background

Cancer is a major cause of death in Europe and worldwide. Nowadays, almost 50% of deaths at middle age is caused by cancer [1]. In addition, socioeconomic inequalities in cancer rates are an important contributor to socioeconomic inequalities in total mortality. During the 1990s, this contribution was large in Southern European countries as reported in several studies [2,3]. This was also reported in other settings where cardiovascular diseases used to play an important role in socioeconomic inequalities in mortality such as Sweden [4] or New Zealand [5], cancer being there now the main driver of socioeconomic inequalities in female mortality during the 1990s.

Time trends in socioeconomic inequalities in cancer mortality differ by cancer site and by country. Large inequalities have been reported for respiratory, cervix uteri, stomach and liver cancer in the US [6]. Over the 1980s and the 1990s, an increase in educational differences in cancer mortality has been shown in the US [7-9] or in Norway [10] for all cancers combined as well as for lung and colorectal cancer, with a decrease in mortality rates more pronounced among higher educated men and women. On the contrary, stable inequalities in cancer mortality have been observed in Barcelona during the 1990s [11]. In France, inequalities in total cancer and specific cancer site mortality increased until the end of the 1990s both in men and women [2]. A specific situation has been observed for breast cancer mortality. Educational differences in mortality disappeared during the 1990s in France [12] as in Finland [13], whereas higher mortality rates among higher educated women were still observed in most countries [14].

These studies focused on the period until the end of the last century, or the very first years of the 2000s. Given that socioeconomic inequalities in cancer mortality within and between countries have changed over time, it is worthwhile examining recent trends especially in a country where large inequalities have been reported in the past. In addition, both relative and absolute measures of inequalities as well as measures comparing the two extreme educational groups and measures taking into account the whole population are needed to get an accurate and comprehensive picture of educational differences in cancer mortality and eventually help the policy makers to tackle these inequalities.

The aim of this analysis is to provide an overview of relative and absolute educational differences in cancer mortality in France during the period 1999–2007 and to compare these inequalities with those reported during the previous decade (1990–1998).

## Methods

The analysis is based on a representative sample of the French population (the Echantillon Démographique Permanent) created by the French National Institute of Statistics (INSEE) containing about 1% of the population [15]. The sample includes all persons born on any one of four specific calendar dates in any year and is regularly updated to include new subjects with any of these birthdays. Data are updated at each successive census. We excluded people born outside of mainland France because their vital status was not adequately recorded. Causes of death were obtained by linkage with the French national death registry (CépiDc, INSERM). The causes of death were identified for over 99% of the deceased included in this analysis. The underlying causes of death were classified according to the International Classification of Disease, Ninth Revision (ICD-9) for the years until 1999 and the International Classification of Diseases, Tenth Revision (ICD-10) for the years 2000–2007.

Socioeconomic status was measured using education level as declared at census in 5 categories: no diploma, primary education, lower secondary or vocational upper secondary education, general upper secondary education, tertiary education.

Educational differences in cancer mortality were studied for the period 1999–2007. In addition, these differences were compared with those observed in the previous decade (1990–1998). Analyses were restricted to people aged 30–74 at 1990 or 1999 census, depending on the analysis. Subjects were followed until death, their 75th birthday, or 31/12/1998 or 31/12/2007 (depending on the analysis), whichever occurred first. We excluded people with missing educational information (n = 156, 0.1% in 1990 and n = 16224, 5.8% in 1999). The analysis was finally conducted among 120,307 men and 130,980 women for the period 1990–1998 and among 127,843 men and 137,833 women for the period 1999–2007.

Analyses were conducted separately for men and women. We used several indicators to assess educational differences in mortality. Relative socioeconomic inequalities in mortality were assessed using Cox regression models, with age as the time variable. We computed hazard ratios (HR) by education as well as relative indices of inequality (RII). Details about the calculation of the RII can be found elsewhere [16]. Briefly, the calculation of the RII is based on a ranked variable for education, which specifies for each educational group the mean proportion of the population with a lower level of education. The RII is then computed by regressing the mortality on this ranked variable. Thus, the RII expresses inequality in the whole socioeconomic continuum. It deviates further from 1 as the educational inequalities in the study population widen. In addition, age standardized mortality rates (MR) were computed with direct standardisation, using the WHO European standard population as standard [17].

A trend test was carried out to test the hypothesis that the RII changed over time. Mortality for the entire period was analyzed using a Cox proportional hazards model, with age as the time variable and the following explanatory variables: the period as a categorical variable to take into account mortality that is generally decreasing over time; the ranked variable for education; an interaction term between the period and the ranked variable for education. This interaction term measures the linear trend of the progression over time of the RII.

We also computed the Population Attributable Fraction (PAF) attributable to education as follows [18]:

$$\mathrm{PAF}=\frac{\sum_{i=1}^{5}p_{i}\left(RR_{i}-1\right)}{\sum_{i=1}^{5}p_{i}RR_{i}}$$

with p_i_ the share of the ith educational group, RR_i_ the hazard ratio of mortality in the ith group when compared with the highest educated. It can be interpreted as the proportion of deaths that could be avoided if all educational groups had the same rate of mortality as the tertiary educated.

Absolute inequalities were assessed with mortality rate difference (RD) between the highest and the lowest education groups. We also calculated the number of deaths attributable to education differences in mortality, thereafter called AD, as the product of the PAF by the average MR [16]. This can be interpreted as the total number of deaths that could be avoided if all educational groups had the same rate of mortality as the tertiary educated.

The research that is reported in the manuscript has been performed with the approval of the CNIL (French data protection agency, reference 902368). The permission to use the data within the frame of this approval has then been given by the organisms in charge of data collection (Insee for census data and Inserm-CepiDc for mortality data).

## Results

Education level increased between 1990 and 1999 both in men and women, the increase was more pronounced among women. In 1999, the education distribution was quite similar among men and women except a higher proportion of women with primary education and a higher proportion of men with lower and vocational upper secondary education (Table 1). Between 1990–1998 and 1999–2007, the age-standardized cancer MR strongly decreased among men from 375 (per 100000) to 312 whereas it remained stable among women (from 161 to 154). In the period 1999–2007, when compared with men with tertiary education, the HR of total cancer mortality was significantly elevated in all the other educational groups, ranging from 1.30 (95% CI: 1.10-1.53) among men with upper secondary general education to 2.40 (2.10-2.73) among men without any diploma (Table 1). Among women, we observed similar significantly higher total cancer MR among the four lower educational groups when compared with tertiary education. During the period 1999–2007, lung, upper aerodigestive tract (UADT) and colorectal cancers were the most frequent cancers among men, and breast, lung and colorectal cancers the most frequent among women. These cancer sites accounted for slightly less than 50% of the total cancer MR both in men and women (Tables 2 and 3).

**Table 1:** Distribution of the population and hazard ratios by education for total cancer mortality during the period 1990–1998 and 1999–2007 among men and women

	**N**	**%**	**N deaths**	**MR**^**1**^	**HR**^**2**^	**95% CI**
MEN						
**1999 - 2007**						
No diploma	20321	15.9	917	442	2.40	2.10-2.73
Primary	19051	14.9	895	345	1.84	1.61-2.10
Lower secondary and vocational upper secondary	52222	40.8	1283	310	1.72	1.52-1.95
General upper secondary	13887	10.9	270	235	1.30	1.10-1.53
Tertiary	22362	17.5	299	186	1	
**1990 - 1998**						
No diploma	26611	22.1	1443	478	2.49	2.15-2.89
Primary	26899	22.4	1332	387	2.02	1.74-2.34
Lower secondary and vocational upper secondary	38051	31.6	972	372	1.95	1.68-2.27
General upper secondary	13453	11.2	328	295	1.54	1.30-1.84
Tertiary	15293	12.7	206	196	1	
WOMEN						
**1999 - 2007**						
No diploma	22741	16.5	425	170	1.41	1.18-1.69
Primary	29020	21.1	621	162	1.36	1.14-1.61
Lower secondary and vocational upper secondary	46108	33.5	633	162	1.40	1.19-1.65
General upper secondary	16618	12.1	195	157	1.33	1.09-1.63
Tertiary	23346	16.9	180	121	1	
**1990 - 1998**						
No diploma	32359	24.7	750	190	1.30	1.07-1.59
Primary	37449	28.6	716	150	1.05	0.86-1.28
Lower secondary and vocational upper secondary	33988	25.9	425	155	1.08	0.88-1.33
General upper secondary	14043	10.7	142	134	0.92	0.72-1.18
Tertiary	13141	10.0	118	146	1	

^1^Age standardized mortality rate, per 100000 person years; ^2^hazard ratio.

**Table 2:** Various measures of educational differences in mortality for total cancer and cancer specific mortality during the period 1990–1998 and 1999–2007 among men aged 30-74

	**N**_**deaths**_	**MR**^**1**^	**RD**^**2**^	**PAF**^**3**^	**AD**^**4**^	**RII**^**5**^	**95% CI**	**p for trend**^**6**^
**1999 - 2007**								
All cancers	3664	312	256	0.40	126	2.42	2.13-2.74	
Lung	1015	86	69	0.38	32	2.45	1.93-3.12	
UADT^7^	427	37	55	0.79	29	5.74	3.91-8.44	
Colorectal	289	24	9	0.15	4	1.52	0.98-2.35	
Liver	212	18	18	0.65	12	3.24	1.89-5.57	
Lymphatic and haematopoietic tissue	209	18	2	0.07	1	1.07	0.65-1.77	
Oesophagus	197	17	26	0.72	12	6.04	3.38-10.8	
Pancreas	174	15	4	0.04	1	1.90	1.07-3.36	
Prostate	189	15	10	0.31	5	1.21	0.71-2.06	
Stomach	102	9	12	0.58	5	3.68	1.69-8.02	
Bladder	111	9	−2	−0.04	0	3.57	1.68-7.57	
Kidney	105	9	10	0.57	5	0.75	0.37-1.50	
Brain and central nervous system	83	8	4	0.19	1	0.95	0.42-2.11	
Other	551	47	39	0.37	17	2.47	1.79-3.42	
**1990 - 1998**								
All cancers	4281	375	282	0.48	180	2.16	1.92-2.43	0.11
Lung	1136	100	83	0.53	52	2.05	1.64-2.57	0.28
UADT^7^	628	58	75	0.82	47	4.79	3.49-6.58	0.34
Colorectal	317	27	5	0.05	1	1.49	0.98-2.27	0.71
Liver	298	25	22	0.56	14	2.32	1.48-3.64	0.31
Lymphatic and haematopoietic tissue	231	20	4	0.14	3	1.31	0.80-2.13	0.75
Oesophagus	248	22	27	0.66	15	4.96	2.96-8.31	0.48
Pancreas	177	15	6	0.39	6	1.19	0.68-2.07	0.22
Prostate	194	16	8	0.46	7	1.23	0.72-2.10	0.70
Stomach	141	12	8	0.45	6	3.36	1.71-6.60	0.98
Bladder	130	11	6	0.55	6	2.04	1.03-4.02	0.39
Kidney	103	9	14	0.83	7	1.37	0.65-2.85	0.16
Brain and central nervous system	96	9	3	−0.11	−1	2.20	1.02-4.74	0.08
Other	582	51	22	0.30	15	1.55	1.14-2.11	0.03

^1^Age standardized mortality rate, per 100000 person years; ^2^Rate difference; ^3^Population attributable fraction; ^4^Number of deaths attributable to differences in education, computed as the product of PAF by MR; ^5^relative index of inequality; ^6^comparison of the RII for the two periods; ^7^UADT = upper aerodigestive tract (lip, oral cavity, pharynx and larynx).

ICD codes: total cancer (140–239 in ICD-9; C00-D47 in ICD-10), and the following cancer sites: UADT (140–149, 161; C00-14, C32), oesophagus (150; C15), stomach (151; C16), colorectal (153–154; C18-C21), liver (155; C22), pancreas (157; C25), lung (162; C33-34), prostate (185; C61), kidney (189; C64-C66, C68), bladder (188;C67), brain and central nervous system (191–192; C70-C72), lymphatic and haemaopoietic tissue (200–208; C81-C96), and other cancers (the rest of 140–239; the rest of C00-D47).

**Table 3:** Various measures of educational differences in mortality for total cancer and cancer specific mortality during the period 1990–1998 and 1999–2007 among women aged 30-74

	**N**_**deaths**_	**MR**^**1**^	**RD**^**2**^	**PAF**^**3**^	**AD**^**4**^	**RII**^**5**^	**95% CI**	**p for trend**^**6**^
**1999 - 2007**								
All cancers	2054	154	49	0.24	37	1.28	1.08-1.52	
Breast	487	38	−2	0.01	1	0.85	0.60-1.20	
Lung	246	19	11	0.40	8	1.49	0.91-2.44	
Colorectal	184	13	10	0.56	7	1.59	0.88-2.85	
Lymphatic and haematopoietic tissue	139	10	5	0.07	1	1.25	0.64-2.44	
Ovary	139	10	0	0.00	0	0.92	0.48-1.77	
Uterus	113	9	6	0.54	5	2.20	1.05-4.58	
Pancreas	114	8	0	0.16	1	0.97	0.47-2.01	
UADT^7^	69	6	6	0.52	3	2.95	1.16-7.48	
Brain and central nervous system	78	6	−2	0.15	1	0.90	0.38-2.16	
Oesophagus	35	3	−1	−0.41	−1	0.90	0.24-3.33	
Stomach	41	3	0	0.36	1	1.21	0.36-4.06	
Kidney	41	3	2	0.31	1	1.99	0.57-6.94	
Liver	40	3	3	0.53	2	3.10	0.85-11.4	
Bladder	20	1	0	−^8^	−^8^	1.90	0.30-12.1	
Other	308	22	12	0.32	7	1.62	1.03-2.54	
**1990 - 1998**								
All cancers	2151	161	44	0.09	15	1.45	1.23-1.72	0.32
Breast	537	42	−3	−0.10	−4	1.19	0.86-1.66	0.38
Lung	155	12	2	0.19	2	0.83	0.45-1.54	0.31
Colorectal	236	17	7	0.15	3	1.66	0.99-2.80	0.93
Lymphatic and haematopoietic tissue	167	12	4	0.21	2	1.32	0.71-2.44	0.83
Ovary	154	12	5	0.35	4	1.18	0.63-2.21	0.89
Uterus	121	9	8	0.58	5	2.77	1.31-5.85	0.26
Pancreas	100	7	8	0.79	6	2.20	0.97-5.02	0.17
UADT^7^	49	4	1	0.02	0	2.83	0.93-8.65	0.93
Brain and central nervous system	70	5	−1	−0.06	0	0.64	0.26-1.59	0.50
Oesophagus	29	2	4	0.45	1	16	2.80-90.9	0.04
Stomach	70	5	7	0.42	2	6.79	2.31-19.9	0.02
Kidney	38	3	−2	−0.66	−2	1.24	0.34-4.46	0.77
Liver	51	3	−1	−0.29	−1	1.31	0.43-4.01	0.62
Bladder	24	2	1	−0.44	−1	1.94	0.37-10.1	0.84
Other	350	26	4	−0.04	−1	1.35	0.89-2.06	0.43

^1^Age standardized mortality rate, per 100000 person years; ^2^Rate difference; ^3^Population attributable fraction; ^4^Number of deaths attributable to differences in education, computed as the product of PAF by MR; ^5^relative index of inequality; ^6^comparison of the RII for the two periods; ^7^UADT = upper aerodigestive tract (lip, oral cavity, pharynx and larynx); ^8^All RR by educational level could not be computed due to too few deaths, therefore no estimation is available.

ICD codes: total cancer (140–239 in ICD-9; C00-D47 in ICD-10), and the following cancer sites: UADT (140–149, 161; C00-14, C32), oesophagus (150; C15), stomach (151; C16), colorectal (153–154; C18-C21), liver (155; C22), pancreas (157; C25), lung (162; C33-34), breast (174; C50), uterus (179–180, 182; C53-C55), ovary (183; C56), kidney (189; C64-C66, C68), bladder (188;C67), brain and central nervous system (191–192; C70-C72), lymphatic and haemaopoietic tissue (200–208; C81-C96), and other cancers (the rest of 140–239; the rest of C00-D47).

During the period 1999–2007, marked relative educational differences in total cancer mortality as measured with RII were observed among men (RII = 2.42, 95% CI: 2.13-2.74) (Table 2). Differences were particularly large for UADT and oesophagus (RII > 5.5). A RII higher than 3 was found for stomach, bladder and liver cancer. An elevated RII of 2.45 was found for lung cancer mortality. On the contrary, no association between education and mortality was found for cancer of kidney, brain and central nervous system, and lymphatic and haematopoietic tissue. Colorectal cancer mortality increased slightly with decreasing education but the RII did not reach statistical significance. For women, modest educational differences in total cancer mortality were found (RII = 1.28, 1.08-1.52) (Table 3). The RII was significantly higher than 1 only for uterus (RII = 2.12, 1.01-4.47) and UADT (RII = 2.95, 1.16-7.48) cancer mortality. High RIIs were found for lung, UADT, colorectal, and liver cancer, but without reaching statistical significance.

When compared with the period 1990–1998, no change was observed in the magnitude of the RII for total cancer and for most specific cancer mortality both in men and women (Tables 2 and 3). A significant increase in inequalities was nevertheless observed for mortality from “other cancers” among men. A decrease was observed among women for mortality from oesophagus and stomach cancers, although based on small numbers. In addition, the RII strongly increased between the two periods for lung cancer among women and a modest increase in RII was reported for total and lung cancer among men, although the estimates did not statistically differ between the two periods. On the contrary, the RII for breast cancer decreased.

If all educational groups had experienced the same mortality as the highest educated, the proportion of cancer deaths avoided (estimated with the PAF) would have decreased among men from 48 to 40% between the 1990s and the 2000s (p for tend = 0.24) but strongly increased among women from 9% to 24% (p for tend = 0.20) (Tables 2 and 3). During the 1999–2007 period, the PAF was highest among men for UADT and oesophagus cancer, followed by liver, stomach, kidney and lung cancer. Among women, the PAF was highest for colorectal cancer, followed by uterus, liver, UADT and lung cancer. It was null for breast cancer. When compared with the previous period, among men the PAF decreased for lung cancer (p for tend = 0.14) and increased for colorectal cancer from 5 to 15% (p for tend = 0.36). Among women, an increase was observed for almost all cancer sites, in particular for colorectal (p for tend = 0.06) and lung (p for tend = 0.23) cancers.

Among men, the RD for total cancer decreased between the two periods (Table 3). The decrease was particularly pronounced for lung and UADT cancers. The total number of deaths avoided if the MR were similar in all educational groups to that among higher educated men (estimated with the AD) would have decreased or remained stable for almost all cancer sites but colorectal cancer. The decrease was more pronounced for AD than for the RD. Among women, the RD between the two periods slightly increased for total cancer, lung, UADT and colorectal cancers but the AD largely increased.

## Discussion

Socioeconomic inequalities in cancer mortality have been observed in different European countries and the US until the end of the 1990s, with contrasted situations between countries and changes over time in the magnitude of these inequalities. To our knowledge, this is the first study documenting socioeconomic inequalities in cancer mortality during the 2000s. It showed substantial relative inequalities in France both for total cancer and specific cancers mortality. When compared to the previous decade, these inequalities tended to remain stable. Important changes were observed for absolute inequalities. In particular, absolute inequalities decreased among men for the most frequent cancers whereas they increased among women for all cancers combined, lung cancer, and especially for colorectal cancer, when assessed with the number of deaths that would be avoided if all women had the same MR as higher educated.

Many measures of inequalities have been defined in the literature [16]. The measures are complementary and allow getting a comprehensive picture of educational inequalities in cancer mortality. Methodological aspects related to the definition of each indicator may partly explain our results. First, the RII quantifies the mean increase in mortality by increasing educational rank. It is therefore less appropriate when there is no gradient between education and mortality, as observed in our data among women for most cancers where the mortality was lowest among highest educated women and similar in most other educational groups. Second, HR or RD only compares two groups whereas RII or AD takes into account the whole population and the relative size and health of each educational group. In particular, when comparing the two periods, the AD and the RD yielded to different results, highlighting the importance of the situation among middle educated people. Among men, the decrease was more pronounced for the AD than for the RD, showing both an improvement of the health among middle educated men combined with a global increase in education. On the contrary, among women, the increase in inequalities was more pronounced for the AD than for the RD for all cancers combined, UADT and colorectal cancers, showing that not only the least educated women but all women experienced a worsening of cancer mortality when compared with the most educated.

Finally, it has been argued that the PAF, and hence AD, may be less appropriate to make comparisons because the size of the reference category may impact the results if it differs between the two populations compared. However, the PAF can be interpreted as the proportional reduction in mortality rates that would occur in the hypothetical and ideal situation where everyone experiences the rate of the highest educated (i.e. the lowest mortality rate) and therefore quantifies the potential for reduction in socioeconomic inequalities. Therefore it provides relevant information from a public health point of view when a major goal of public health policies is to tackle health inequalities.

As we are describing educational inequalities in the French population in two periods, there is an overlap between the two samples analyzed, and the large majority of the 1999 population was included in the 1990 population. Therefore the dramatic change in educational attainment between 1990 and 1999, especially among women, represents a lower qualified older age group being replaced by a higher qualified younger age group. This change, however, does not explain our findings. Indeed, analyses conducted among the younger women in the first period, or excluding the women that entered the sample in the second period lead to similar results (results not shown).

During the 2000s, the largest relative educational differences were reported for lung, UADT, oesophagus, pancreas, and bladder cancer, all these cancers being associated with smoking [19]. Inequalities were particularly large for respiratory cancers both in men and women. When compared with the previous decade, these inequalities increased among women and tended to be stable among men. However, both in men and women, they are likely to increase in future years. Indeed, socioeconomic inequalities in smoking have increased during the last decades both among women and men in France [20,21]. Moreover, an increase in educational differences in lung cancer mortality has been reported in the younger generations during the 2000s [22]. On the contrary, diverging trends between men and women are observed with regards to absolute inequalities. Among men indeed, absolute inequalities in smoking and/or alcohol related cancers, namely lung, UADT and oesophagus [19,23], decreased during the 2000s whereas the available evidence suggested an increase until the end of the 1990s [24]. Among women on the other hand, absolute inequalities as measured with RD or AD have increased during the last decade and are expected to increase further, due to the large increase in smoking rates [25]. Smoking is still and will remain a large contributor to socioeconomic inequalities in cancer mortality. Studies have consistently pointed to the difficulty to implement efficient policies aiming at reducing social inequalities in smoking [26]. However, even if we manage to reduce both smoking and socioeconomic inequalities in smoking, it will take decades for relative inequalities in smoking related cancers mortality, especially lung cancer mortality, to decrease [27].

Alcohol consumption accounts for a part of cancer mortality including head and neck, oesophagus and liver. A large decrease (approximately 30%) in alcohol consumption has been observed from 1960 to 2001 [28]. Literature on socioeconomic differences in alcohol consumption is sparse, and to our knowledge, there is no study on time trends in these differences. However one study reported that around 1990 in France inequalities were found in excessive alcohol consumption [29].

We observed modest and stable over time relative inequalities in colorectal cancer mortality. A similar pattern was reported in Barcelona [11,30] but it contrasted with the large inequalities found in the US [6]. Educational differences in the prevalence of overweight or obesity, one of the main risk factor for colorectal cancer [31], may explain these international differences. In France, both rates and educational differences in obesity have increased between 1991 and 2003, especially among women [32]. As a consequence, educational differences in colorectal cancer incidence rates are likely to increase in future years. We already observed an increase in several inequality measures between the 1990s and the 2000s. First, colorectal cancer is the only frequent cancer where the PAF increased between the 1990s and the 2000s, especially among women. In addition, the MR decreased between the two periods, but absolute inequalities as measured by RD or AD increased, especially among women. Moreover, a nationwide screening is being implemented in France. The screening rate is still low, around 40% [33], but higher among people with higher socioeconomic position (SEP) [34]. In order not to increase social inequalities in colorectal cancer survival, public health policies should devote special efforts to increase screening rates in all social groups. Although this had not been observed until now, colorectal cancer may become a large contributor to social inequalities in cancer mortality in the coming years in France.

In France, educational differences in breast cancer mortality have disappeared during the 1990s [12]. This trend was also observed in Finland [13] and in other European countries among younger women [14]. Our results show that the lack of association between education and breast cancer mortality seems to remain during the 2000s both on the relative and absolute scale. Trends in socioeconomic inequalities in breast cancer mortality are difficult to assess, in particular because these inequalities combine inequalities in incidence that favour women with a lower SEP [35] and inequalities in survival that favour women with a higher SEP [36]. Breast cancer is a multifactorial disease, however age at first birth is suggested to be the main risk factor explaining social differences in breast cancer incidence [37-39]. Literature suggests diminishing differences in age at first birth between educational groups over time, with a postponement of age at first birth among lower educated women [40]. A nationwide screening for breast cancer has been implemented in France at the beginning of the 2000s and is likely to impact socioeconomic inequalities in cancer survival. Socioeconomic inequalities in screening uptake are still reported in France [41]. However, a recent study showed an increase in screening rates in all socioeconomic groups between 2000 and 2005, and as a consequence, a decrease in absolute difference in screening rates between women with the highest and the lowest SEP [42]. Therefore, inequalities in breast cancer mortality are not expected to largely change in the coming years.

Some methodological issues should be discussed. Our analysis was based on a large sample representative of the French population born in mainland France. The population born in French overseas territories was excluded because the causes of death were not adequately recorded over the follow-up period for this population. In addition, we limited our analyses to people aged below 75 due to the less accurate certification of causes of death among older subjects. A few limits in the codification of causes of death should be mentioned. For uterine cancers, tumours of the endometrium or cervix could not be distinguished because 45% of uterine deaths were coded ‘Malignant neoplasm of uterus, part unspecified’. However, the educational differences observed in uterine cancer mortality are mostly driven by cervical cancer [43]. Misclassification of some secondary cancers as primary liver cancers is also likely to have occurred. The percentage of missing values on education was extremely low in 1990 due to some internal procedures performed by Insee on the 1990 census dataset. This could have slightly altered the results; however we expect the influence, if any, to be small. Conversely, 5.8% of our population had missing education in 1999. Additional analyses showed that this group displayed mortality similar to that found among men and women with primary education.

## Conclusion

Cancer remains a major contributor to socioeconomic inequalities in mortality in France. The reduction of social inequalities in cancer is one of the main public health policy targets of the French Cancer Plan 2009–2013. In this regard, this study provides important results, documenting areas of improvement during the last decade and those where progress is still needed. Relative inequalities remained globally stable among men and women, but the situation regarding absolute inequalities differed by gender. Among men, an important decrease was observed during the 2000s, especially for several frequent cancers (lung, UADT and oesophagus), whereas inequalities seemed to increase for colorectal cancer. In contrast, among women, although the lack of inequalities for breast cancer persisted during the 2000s, the situation regarding social inequalities is less favourable, especially for colorectal cancer, mainly due to a health improvement limited to higher educated women.

## Competing interests

The authors declare that they have no competing interests.

## Authors’ contributions

GM, GR, EJ and DL participated in the design of the study. GM carried out the analyses and drafted the manuscript. GR helped in the statistical analyses. All authors discussed the analysis and the results, helped to draft the manuscript, read an approved the final manuscript.

## Pre-publication history

The pre-publication history for this paper can be accessed here:

http://www.biomedcentral.com/1471-2458/13/823/prepub
